# Paleoenvironmental changes in the coastal zone of the northwest South China Sea during the last 13 kyr

**DOI:** 10.1038/s41598-023-40721-5

**Published:** 2023-08-19

**Authors:** Dingyong Liang, Shuzhuang Wu, Guoqiang Xu, Changjian Xia, Fanglei Gao, Yihua Lin, Juan Du, Liyun Jia

**Affiliations:** 1 The Key Laboratory of Marine Geological Resources and Environment of Hainan Province, Haikou, 570206 China; 2 Hainan Geological Survey, Haikou, 570206 China; 3 Institute of Earth Sciences, University of Lausanne, 1015 Lausanne, Switzerland; 4 Sanya Exploration Institute of Hydrogeology and Engineering Geology, Sanya, 570206 China; 5 Institute of Geomechanics, Chinese Academy of Geological Sciences, Beijing, 100081 China; 6 Comprehensive Institute of Geological Investigation of Hainan Province, Haikou, 570206 China

**Keywords:** Ocean sciences, Planetary science

## Abstract

Marine sediments in coastal zones serve as valuable archives for understanding the history of silicate chemical weathering and summer monsoon rainfall in source areas, providing insights into terrigenous climate and environmental evolution. In this study, we investigated the grain size, clay minerals, and geochemistry of sediments retrieved from core KZK01 in the coastal zone of the northwest South China Sea during the past 13 thousand years before present (kyr BP). Our findings demonstrated that the illite crystallinity index served as a reliable proxy for assessing the intensity of chemical weathering in the source area. Moreover, it distinctly recorded significant climatic events such as the Younger Dryas and Bond events during the Holocene. The dominant driver of the regional East Asian summer monsoon was identified as summer solar radiation in the Northern Hemisphere at low latitudes. Cold climate events exhibited global consistency, potentially influenced by the presence of ice sheets at high latitudes. Lastly, our records revealed a distinct transition at 9.0 kyr, highlighting significant impacts of the Qiongzhou Strait and sea level rise on regional climate dynamics.

## Introduction

**Figure 1:**
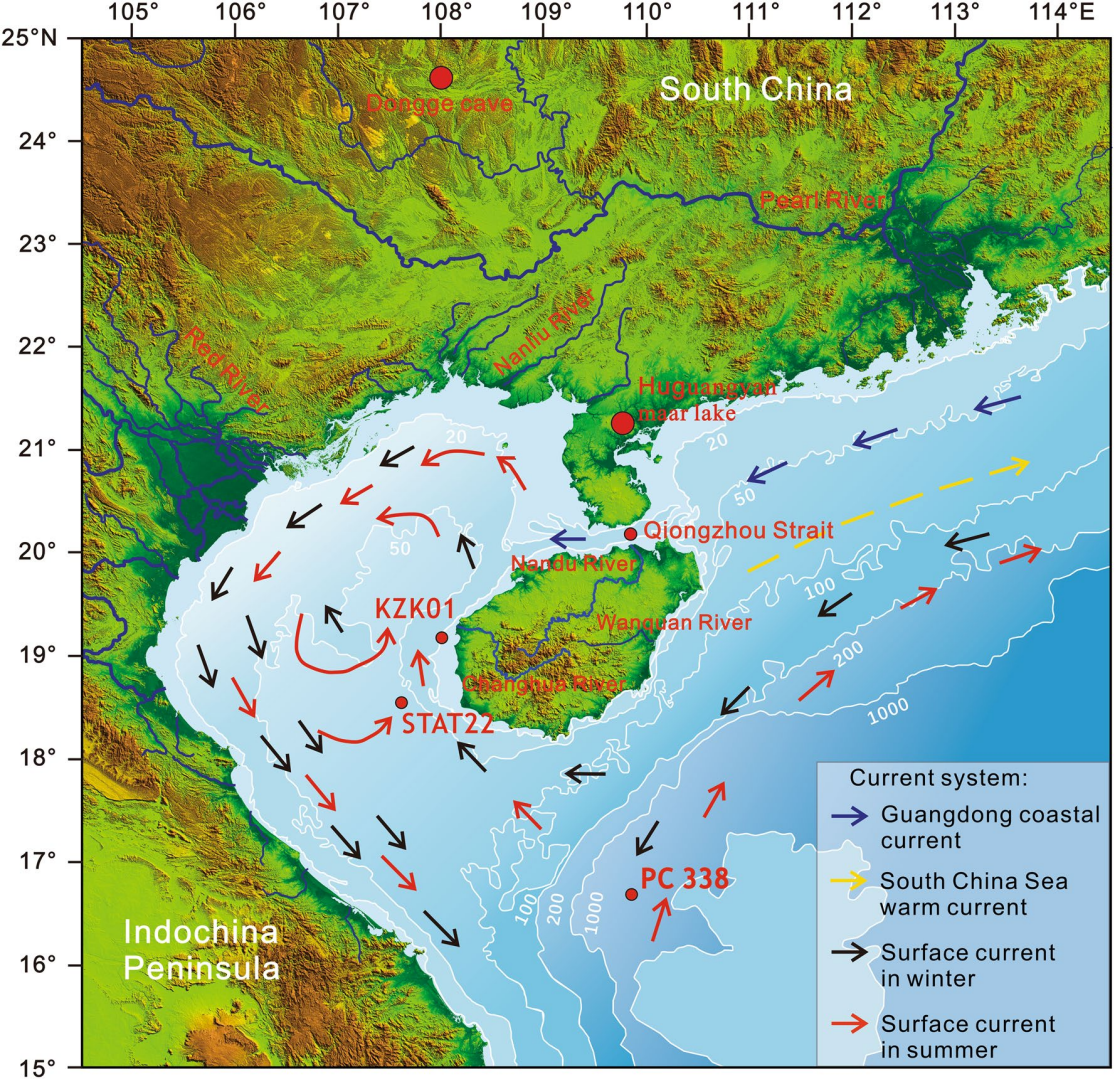
Topography of the area around Western Hainan Island, fluvial systems, and ocean current systems around the continental landmass. The figure shows the positions of core KZK01, core STAT22^18^, core PC338^11^, and the Dongge Cave^24^, which are investigated in this work. It also shows the winter and summer ocean currents in the Beibu Gulf^25–28^, Vietnam coastal current data^29^, coastal current data from western Guangdong^30^, South China Sea warm current^31^, and the surface currents in the South China Sea^32,33^.

The sea-land interaction in the coastal zone is dynamic and highly sensitive; it not only records past transgression history, coastal environmental evolution, and sea level changes, but also yields information on climate fluctuations, ocean and river changes, ecological environmental evolution, and anthropogenic impacts on the environment^1^. Coastal zone deposition plays a pivotal role in preserving paleoenvironmental change records, thereby serving as a critical component within the broader "source" to "sink" system of the continental margin^2^; thus, it has attracted considerable academic interest in the field of geoscience in recent years. The western sea area of Hainan Island is a semi-closed shallow bay (Fig. 1). This bay receives terrigenous clastic materials from sources such as Hainan, the Qiongzhou Strait, Guangxi, Honghe River, and the coastal rivers of Vietnam. The rate of deposition of these materials is high, which provides favorable conditions for the study of climatic events on a centennial time scale^3^, and this region is an excellent research area for the reconstruction of the evolution of the East Asian monsoon^4–6^. Global climate change arises from the combined influences of natural factors and human activities. According to the Intergovernmental Panel on Climate Change (IPCC), the exponential increase in human activities accounts for 90% of the increase in the greenhouse effect. Climate changes trigger extreme weather events like heavy rainfall, floods, tsunamis, and rising temperatures, which severely threaten human activities and economic development^7^. The evolution of climate during the late Quaternary holds crucial implications for future challenges faced by modern humans. Therefore, it is particularly essential to study the environmental evolution of coastal zones since the late Quaternary^8^.

The geochemical characteristics of marine sediments record the history of silicate chemical weathering and summer monsoon rainfall in the source area and provide information on the evolution of the terrigenous climate and environment^9,10^. Li et al.^11^ performed principal component (PC) analysis using geochemical element data from core PC338 in the Qiongdongnan Basin in the northwestern part of the South China Sea. They found that the Red River Basin is controlled by the Indian Summer monsoon^11^. Xu et al.^12^ found a good correlation between the chemical weathering index of the near-CS11 core in the northern deep basin of the South China Sea and worldwide cold events (such as YD, H1, H2, and H3), indicating that the weathering intensity of sediments in this region was mainly controlled by global climate change on a multi-centennial time scale^12^. Previous researchers have conducted extensive paleoclimate reconstruction work around Hainan Island, primarily focusing on the continental shelf and semi-deep sea areas, which are characterized by stable sedimentary environments^13,14^. However, there has been a relative scarcity of studies specifically targeting the coastal zone, which offers proximity to the land and convenient sample collection. It is crucial to determine whether coastal sediments hold comparable significance to deep-water sediments in documenting paleoclimate. Furthermore, identifying effective proxies that serve as indicators of chemical weathering in coastal sediments is equally important.

The Qiongzhou Strait is located between Leizhou Peninsula and Hainan Island. It is approximately 80 km long from east to west and 19 km wide from north to south, with a maximum depth of 120 m. It is one of the three major straits in China. The time of formation of the Qiongzhou Strait has remained a matter of debate. Based on a systematic study of regional geology, geophysics, marine hydrology, regional paleobiogeography, and other data, Zhao et al. concluded that the Qiongzhou Strait formed between 10,570 ± 560 and 7125 ± 96 a BP from the original lowland in the submerged gorge area of the middle Holocene global transgressions^15^. Through the interpretation of high-resolution shallow stratigraphic profiles at the east and west entrances of the Qiongzhou Strait and the use of regional drilling age data, Ni et al. indirectly inferred that the Qiongzhou Strait opened fully at approximately 8.0 kyr BP, which was attributed to the Holocene transgression in the area^16,17^. The provenance of the sediments in core STAT22, which are similar to the sediments in the target area of the present study, has been extensively investigated in previous studies, and it is considered that the sediments in this area mainly originated from the west of Hainan Island before 4500 a BP^18^. After 4.5 kyr BP, with the opening of the Qiongzhou Strait, the provenance and hydrodynamic conditions underwent significant changes, and the area began to receive deposits from sediments in the eastern part of the Qiongzhou Strait. Through geophysical exploration, geological sampling, and numerical simulation, a new understanding of the genesis and formation age of the Qiongzhou Strait has been obtained. However, studies on the formation of the Qiongzhou Strait are still limited by the lack of direct evidence related to high-precision petrology, geochemistry, and chronology.

In this study, core KZK01 from the northwestern coastal zone of Hainan Island was investigated to evaluate the feasibility of using this part of the island as a reconstruction site for the East Asian monsoon, and the changes in sedimentary records before and after the opening of the Qiongzhou Strait were discussed. The sediment granularity, clay minerals, and element geochemistry were analyzed using accelerator mass spectrometry (AMS) ^14^C and optically stimulated luminescence (OSL) dating, and the characteristics of the sediments in the northwestern coastal zone of Hainan Island since 13 kyr BP were described. A proxy index of monsoon intensity suitable for coastal zones reflecting regional surface chemical weathering was selected to explore the element record and the mechanism driving the evolution of the regional summer monsoon and to thereby elucidate the climate change process in the northwestern part of the South China Sea since 13 kyr BP. The results of this study are also expected to provide evidence for the understanding of major regional geological events, such as the formation and evolution of the Qiongzhou Strait.

## Study area

The study area is located in the northwestern part of the South China Sea and is bounded by Hainan to the east, Guangxi to the north, Vietnam to the west, the hinterland of the South China Sea to the south, and the northeastern part of the South China Sea through the Qiongzhou Strait. The depth of the seabed gradually increases from north to south, with the isobaths being approximately parallel to the coastline. The water depth in the western part of the coring site ranges from 20 to 60 m, with an average depth of 40 m and a maximum depth of 106 m (Fig. 1)^19^. Numerous rivers empty into the South China Sea along its northern shore, among which the Red River is the most important sediment source, transporting approximately 125 × 10^6^ t of terrigenous particulate matter to the Beibu Gulf annually^20^. In addition, the South China Sea area also receives sediments from rivers along the coast of Vietnam, Hainan, and Guangxi, and the Pearl River also provides a certain amount of terrigenous material to the northeastern part of the Beibu Gulf through the Qiongzhou Strait^21–23^.

The land area around the study region consists of Proterozoic to late Paleozoic calc-magnesium carbonate rocks, quartzite, metamorphic sandstone, slate, Mesozoic (Jurassic–Cretaceous) continental clastic rocks, Hercynian to Indochinese granite, granite porphyry, quaternary basalt, and loose sediments^19^. The study area has a tropical and subtropical maritime monsoon climate, which is mainly controlled by the monsoon at low latitudes in East Asia. The southwest monsoon prevails in summer and the northeast monsoon prevails in winter. The circulation in the study area exhibits a consistent counterclockwise pattern throughout the year, influenced by the East Asian monsoon, ocean currents, sea temperature variations, and topography (Fig. 1)^25–27^. The water desalination of the Red River keeps the Vietnam coastal current flowing from north to south throughout the year^29,30^. Fluvial sediments originating in South China are carried by the northeast-southwest oriented coastal flow through the Qiongzhou Strait and are integrated into the counterclockwise circulation^31^. In addition, the South China Sea warm current occurs to the northeast of Hainan Island; it flows to the northeast of the South China Sea along the 100 m isobath throughout the year^28^.

## Materials and methods

**Figure 2:**
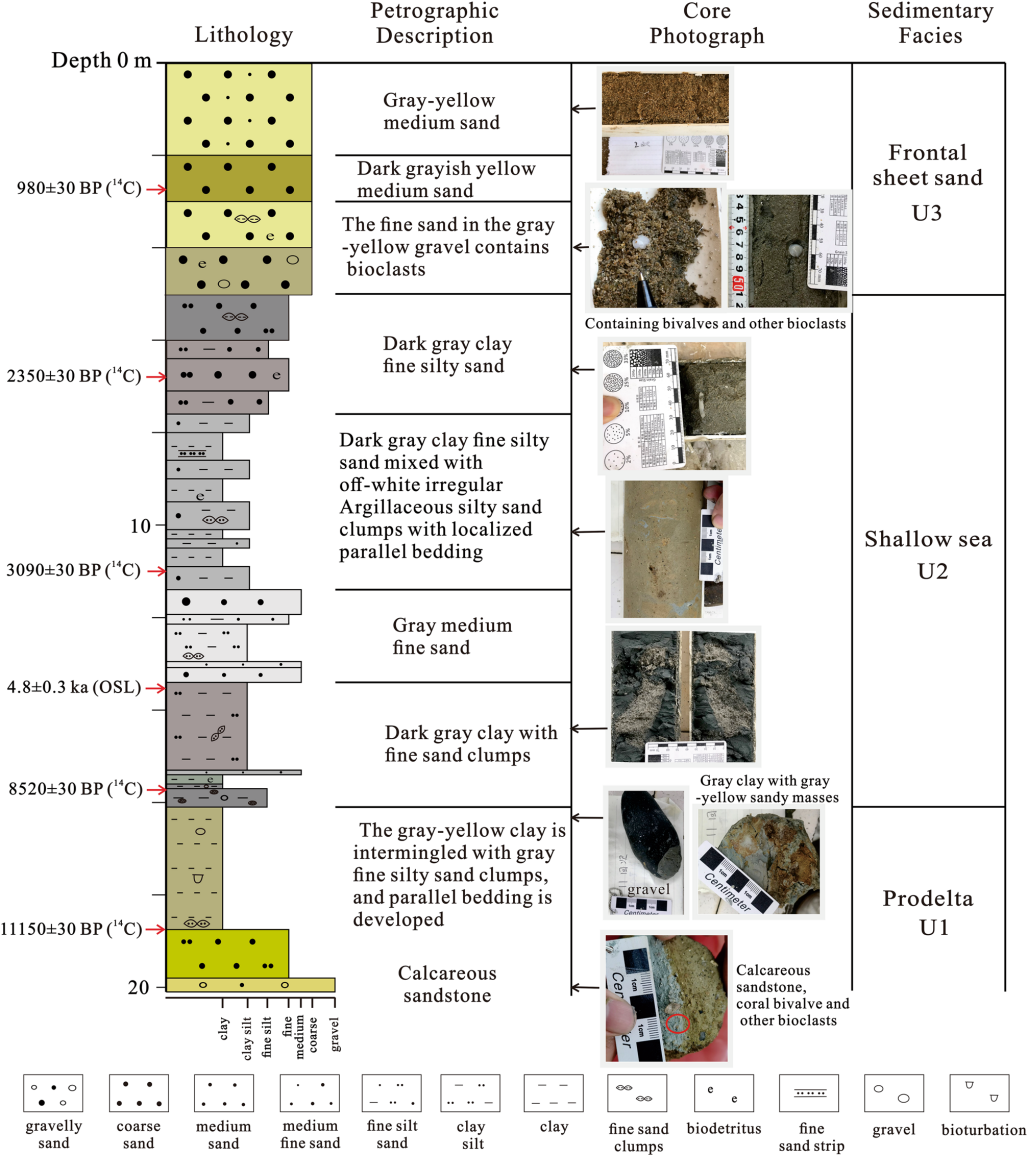
Lithological description, AMS ^14^C ages, OSL ages, and sedimentary facies of core KZK01.

Sediment core KZK01 (19°12′58.08″ N, 108°33′05.78″ E; Fig. 1) was obtained in April 2020 at the mouth of the Changhua River via mechanical rotary coring. The water depth was 12.6 m, the total length of the core was 20.1 m, and the coring rate was 95%. The sediments in the core mainly consisted of interbedded sand and mud, and the specific lithologic characteristics are shown in Fig. 2. In all, 48 samples were taken from the whole tube core from 30 to 40 cm interval, and the particle size, major and trace elements, rare earth elements, and clay minerals were analyzed at the Hainan Provincial Key Laboratory of Marine Geological Resources and Environment.

Particle size was analyzed according to GB/T 12763.8.6.3-200+7. Hydrogen peroxide (10 ml) was added to 10–20 g of samples to remove organic matter. Then, 15 ml of 15% acetic acid was added to remove carbonates from the sample. The samples were boiled with 300 ml of (NaPO_3_)_6_, cooled, and placed on a shaking table for 24 h. The samples were tested using a laser particle size analyzer (UltimaIV-185). The grain resolution was 0.01Φ, the measurement range was 0.02–2000 μm, and the relative error of repeated measurement was less than 1%.

Major and trace rare earth element analyses were conducted in accordance with the GB/T20260.8-2006 standard. Major element analysis was conducted using X-ray fluorescence spectrometry (XRF; ZSX-Primus II), and the mass loss during combustion was determined using gravimetry. Rare earth and trace element analysis was performed using inductively coupled plasma mass spectrometry (ICP-MS; Thermo Field iCAP Qc).

Clay minerals were analyzed using clay-size (< 2 μm) components by X-ray diffraction (XRD). Distilled water and H_2_O_2_ were used to remove salt and organic matter from about 10 g of sediment samples. Then, an appropriate amount of distilled water and (NaPO_3_)_6_ was added, and the mixture was stirred thoroughly and allowed to stand for 2 h. Based on the sedimentation time specified by Stoke’s principle, the upper suspended particles smaller than 2 μm were extracted using needles and centrifuged for concentration treatment. Oriented slices were obtained using the smear method and air dried at room temperature. The natural sheet was directly subjected to diffraction testing and then steam-treated with ethylene glycol for testing (ethylene glycol sheet). A Rigaku Ultima IV-185 diffraction analyzer was used for XRD analysis. The working voltage was 40 kV and the working current was 40 mA. Continuous step scanning was employed. The scanning step width was 0.02°, the scanning speed was 10°/min, and the scanning range was 2.5° to 65°. The quantitative analysis method was the K-value method.

Clay minerals were identified and interpreted using XRD patterns obtained from the three main directional slices. Semi-quantitative calculation of the crest parameters was performed using Jade 6 software. The relative content of the clay minerals was mainly determined based on the ratio of the diffraction peak area of the crystal plane, smectite was determined based on the 1.7 nm (001) crystal plane, and illite was determined based on the 1 nm (001) crystal plane. Kaolinite (001) and chlorite (002) were determined based on the 0.7 nm superimposed peak. The contents of clay minerals, smectite, and illite were calculated by multiplying by the weight coefficients of 1, 4, and 2, respectively. The content of kaolinite and chlorite was determined by fitting the ratio of the peak area between 0.357 and 0.354 nm. The crystallinity index of illite was expressed using the full-width at half-maximum (FWHM) of the diffraction peak at 10 Å on the curve. The crystallinity index was negatively correlated with the crystallinity^34^.

**Figure 3:**
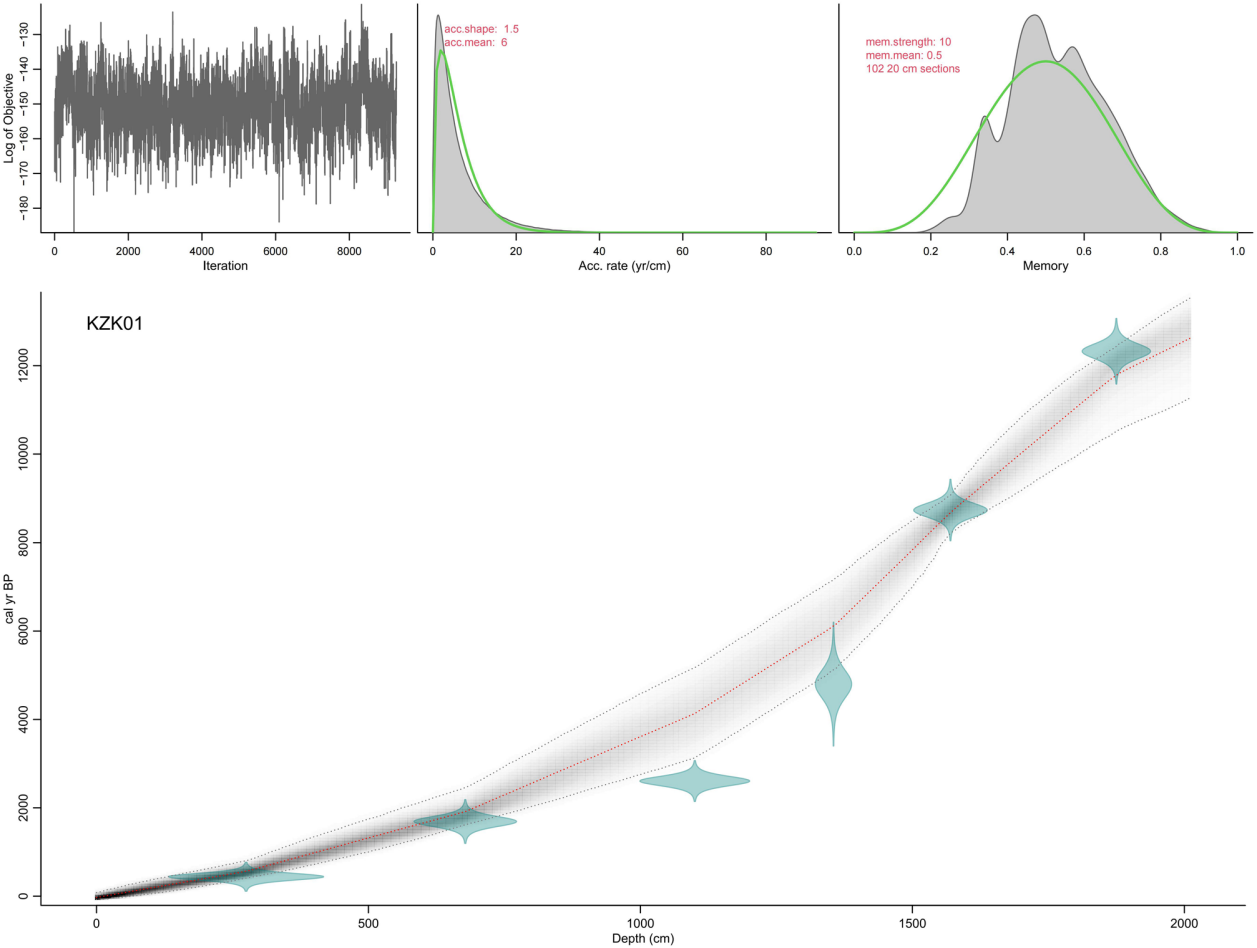
Age-depth model of core KZK01. The upper panels depict the MCMC iterations (left), the prior (green curves) and posterior (grey histograms) distributions for the accumulation rate (middle panel) and memory (right panel). The bottom panel shows the age-depth model, aurantiacus indicates more likely calendar ages, black stippled lines show 95% confidence intervals, and the red stippled curve shows the single ‘best’ model based on the average age for each depth.

In all, six dating samples were collected from core KZK01. ^14^C dating samples were collected from relatively intact shells, foraminifera, and organic matter in the core. ^14^C measurements were carried out by accelerator mass spectrometry (AMS) in Beta Laboratory, USA (Table 1). The AMS ^14^C ages were calibrated to calendar ages using the MARINE 20 calibration curve^35^. We adopted the average reservoir age (− 15 ± 38 years) around the South China Sea for the last thousand years^36–39^. Yu et al. revealed that significant fluctuations in ^14^C marine reservoir ages and regional marine reservoir corrections occurred in the South China Sea during the Holocence^40^. Therefore, reservoir age corrections of 89 ± 59 years and 151 ± 85 years were applied for the periods 2–3.5 kyr and 3.5–13 kyr BP, respectively. The Bayesian age-depth model of core KZK01 was calculated using the R program BACON^41^ (Fig. 3). The OSL dating sample was obtained from the fine sand layers, and the experiment was carried out using a Riso TL/OSL-DA-20 thermoluminescent/optometric instrument at the Laboratory of the Three Gorges Research Center for Geological Hazards of the Yangtze River, Ministry of Education, China University of Geosciences (Wuhan). The sample was preheated at 260 °C for 10 s, and the experimental dose was preheated at 220 °C for 10 s. A 90% blue light emitting diode (λ = 470 ± 20 nm) was used as the excitation light source. The sample was illuminated with blue light for 40 s at 130 °C, and the luminescent signal was recorded through a Hoya U-340 filter with a thickness of 7.5 mm into a 9235QA photomultiplier. The irradiation source was a ^90^Sr/^90^Y β source. The purity of quartz was measured using infrared (IR) light. The results showed that the infrared luminescence signals of feldspar of the two samples were very low, and the IRSL/OSL value was less than 10%. The equivalent dose was measured using the SAR-SGC method combined with the SAR method (Table 1).

**Table 1:** AMS ^14^C and OSL dating of core KZK01.

Sample umber	Dating method	Sampling position/ m	Material	δ^13^C/‰	δ^18^O/‰	Conventional radiocarbon age/a BP	Corrected age/cal. yr BP		
							Range (1σ)	Range (2σ)	Median
KZK01-C1	AMS ^14^C	2.75	Shell	− 1.6	− 4.9	980 ± 30	370–514	283–563	440
KZK01-C3	AMS ^14^C	6.78	Shell	1.6	− 2.4	2350 ± 30	1584–1793	1492–1907	1690
KZK01-YK6	AMS ^14^C	11	Foraminifera	− 0.7	− 3	3090 ± 30	2507–2718	2365–2782	2606
KZK01-G02	OSL	13.55	Quartz			4800 ± 300		4800 ± 300	
KZK01-C4	AMS ^14^C	15.7	Shell	0.2	− 2.7	8520 ± 30	8589–8885	8456–9000	8737
KZK01-YK18	AMS ^14^C	18.75	Organic matter	− 22.4		11,150 ± 30	12,168–12,487	11,999–12,603	12,327

## Results

**Figure 4:**
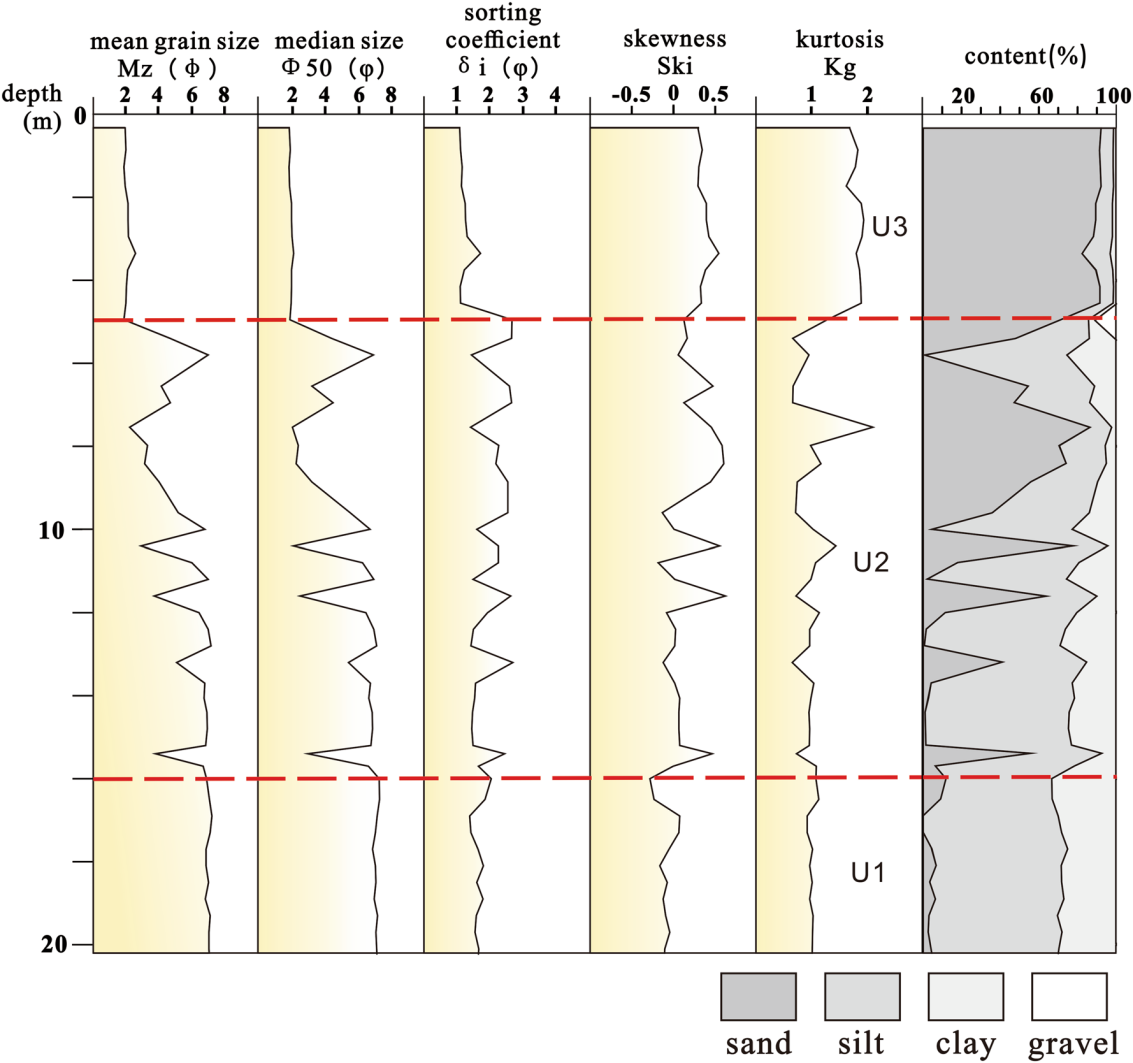
Vertical distribution of grain size composition of core KZK01.

Particle size analysis reveals that the sediment samples are mainly composed of fine medium sand, medium fine sand, and muddy fine silty sand; particle sizes range from 1.93 to 7.23φ, with an average of 4.95φ (Fig. 4). The separation coefficients range from 1.07 to 2.68φ, with an average of 1.74φ. The skewness ranges from − 0.28 to 0.63, with an average of 0.16. The peak state ranges from 0.65 to 2.11, with an average of 1.19. The median diameter ranges from 1.87 to 7.32φ, with an average of 4.81φ.

The predominant clay mineral in the study area is illite, accounting for a relative content ranging from 59 to 93%. Following illite, kaolinite is the next most abundant clay mineral, with an average content of 10.66%. The average contents of chlorite and smectite are 8.44% and 7.0%, respectively.

The average contents of SiO_2_, Al_2_O_3_, Fe_2_O_3_, CaO, MgO, Na_2_O, K_2_O, TiO_2_, P_2_O_5_, and MnO are 75.92%, 11.71%, 3.14%, 1.89%, 1.16%, 1.09%, 3.71%, 0.51%, 0.06%, and 0.06%, respectively. The average contents of Cr, Co, Ni, Cu, Zn, Nb, Ta, Hf, Zr, and Ce are 45.45 mg/kg, 8.08 mg/kg, 17.19 mg/kg, 14.66 mg/kg, 51.05 mg/kg, 13.84 mg/kg, 1.68 mg/kg, 4.38 mg/kg, 132.45 mg/kg, and 54.51 mg/kg, respectively.

**Figure 5:**
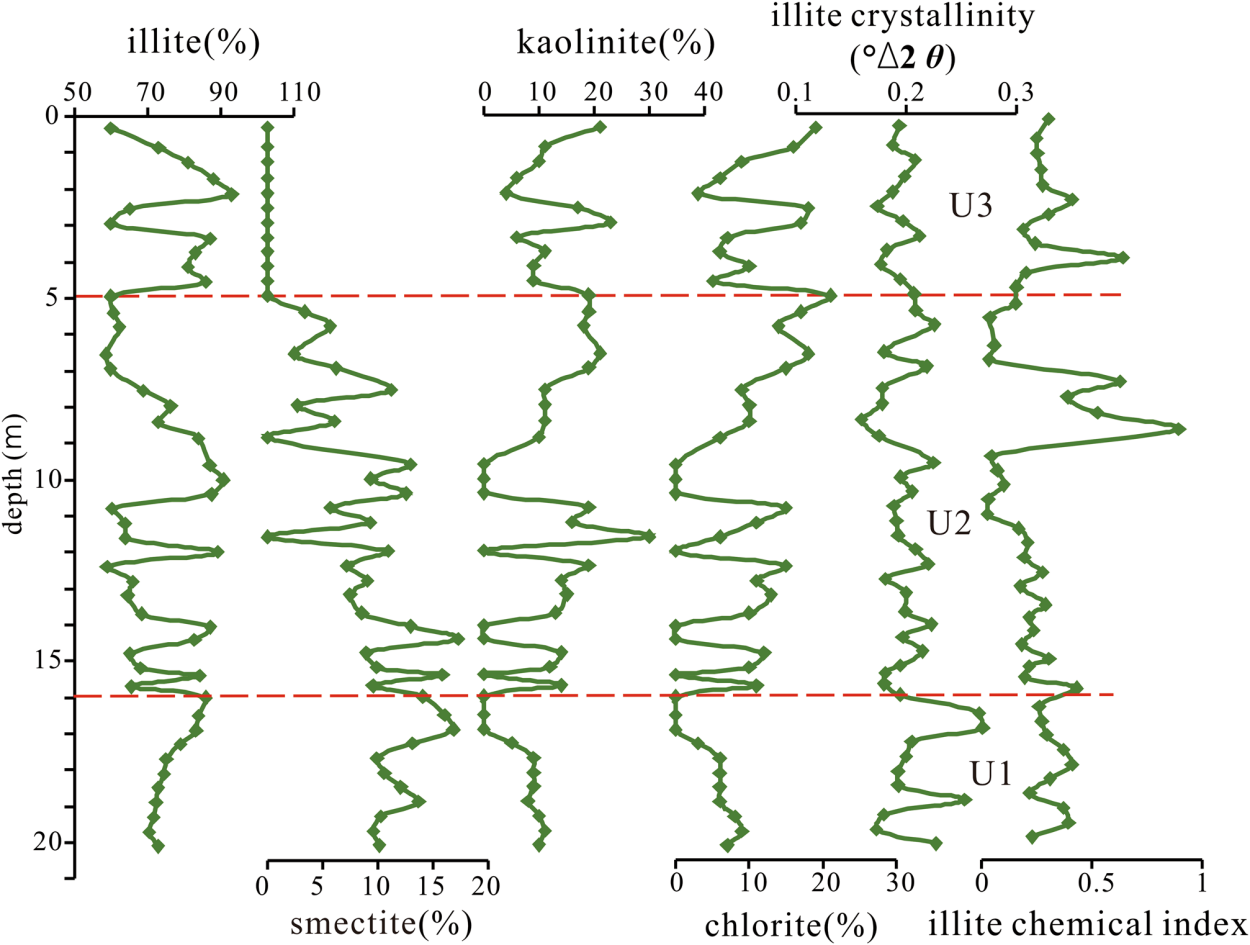
Vertical distribution of clay mineral content from core KZK01.

**Figure 6:**
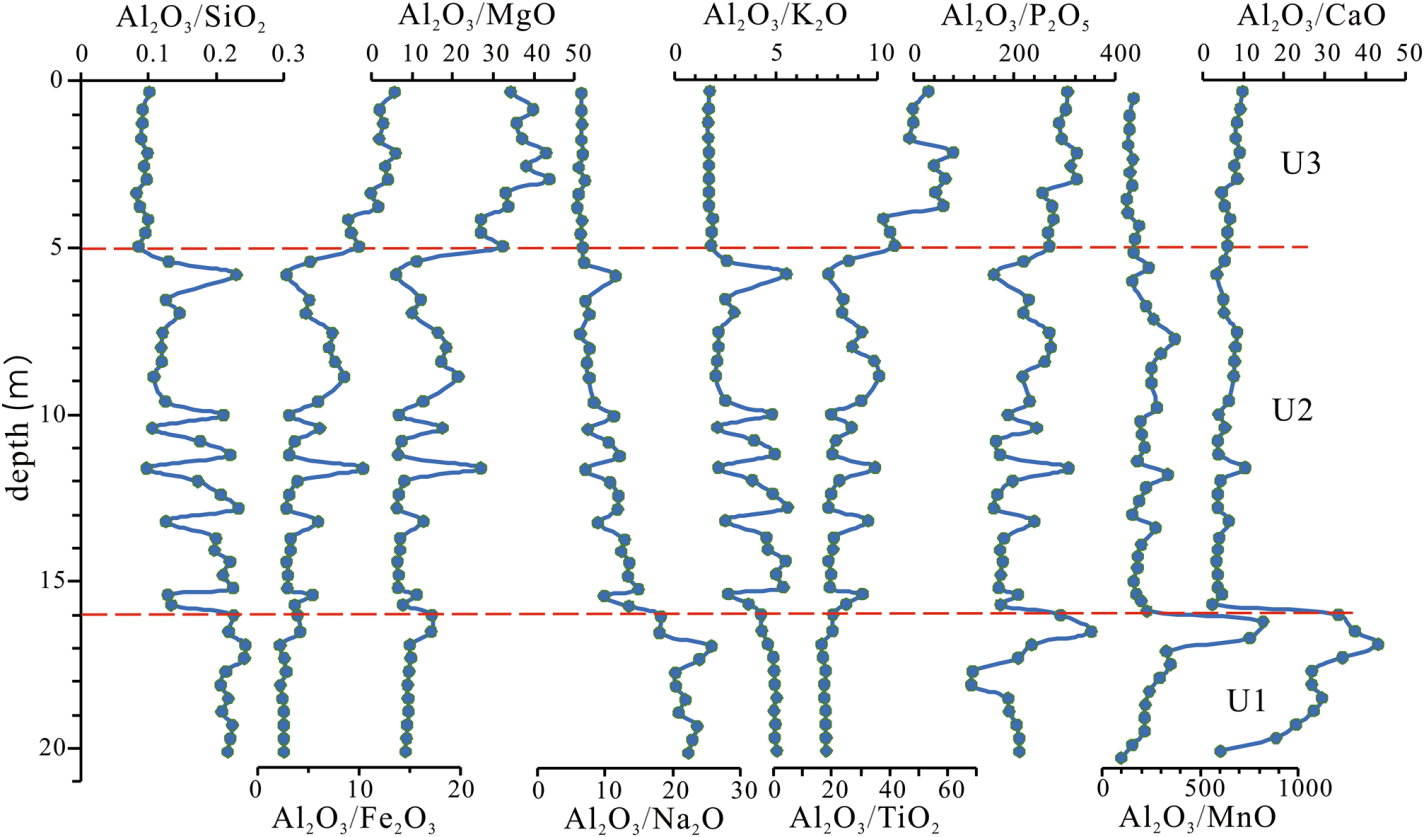
Vertical distribution of major elements from core KZK01.

**Figure 7:**
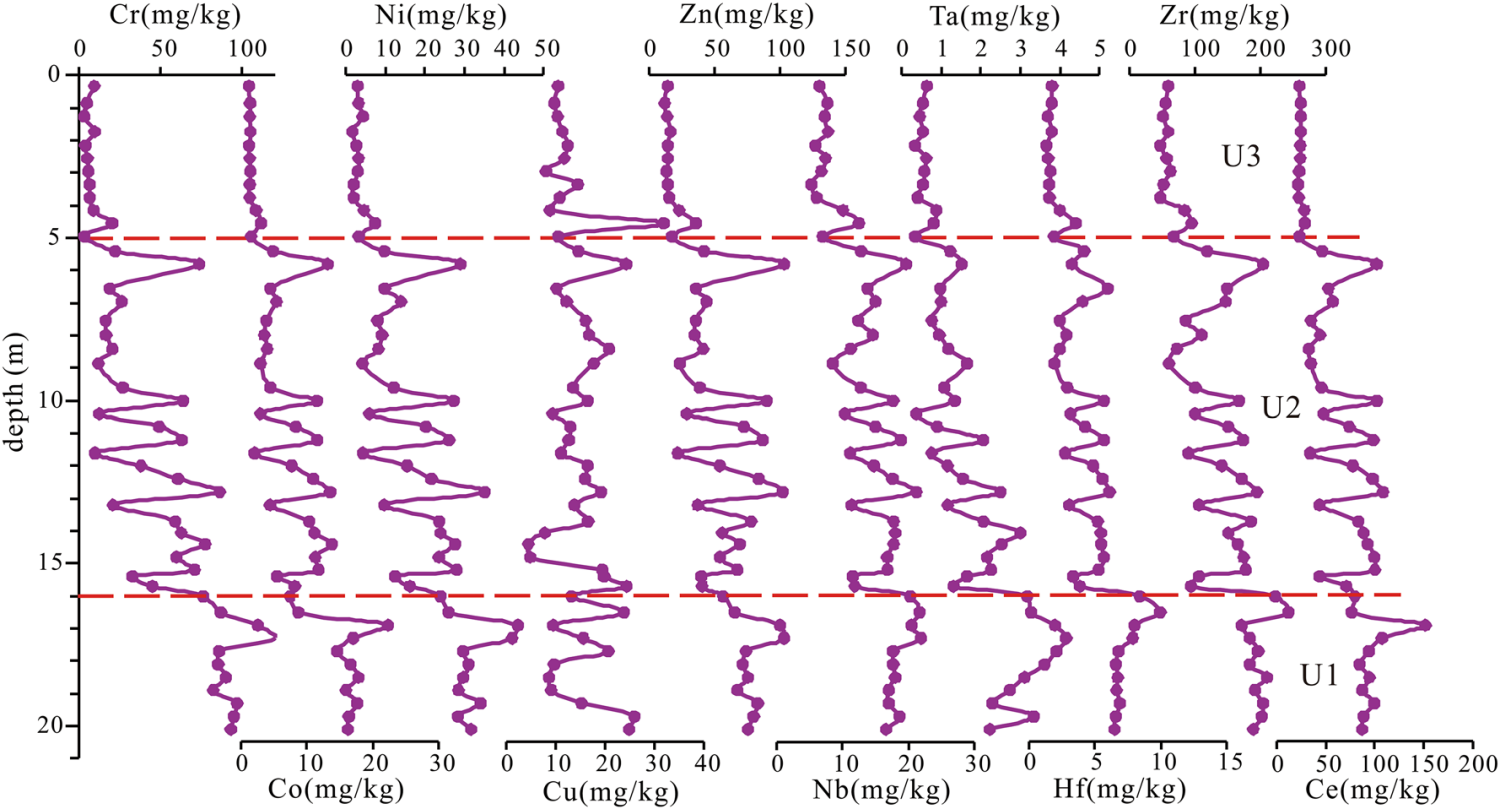
Vertical distribution of trace elements from core KZK01.

Based on particle size, clay minerals, and major and trace elements, the core can be divided into the following three sedimentary units (Figs. 4, 5, 6, 7).

U1 (20–16 m): The sediments are dominated by gray-yellow clay, partially containing fine gray silt clumps; parallel bedding is developed. In this unit, the contents of illite and smectite gradually increased, whereas those of kaolinite and chlorite decrease. The crystallinity of illite shows a gradually increasing trend, and the illite petrochemical index does not change much. The ratios of major elements Al_2_O_3_/P_2_O_5_ and Al_2_O_3_/MnO show an increasing trend from bottom to top, whereas that of Al_2_O_3_/Na_2_O shows a decreasing trend. The ratios of other major elements do not change much from bottom to top.

U2 (16–5 m): The sediments are mainly gray-black clay and silty sand, with an average particle diameter of 5.48Φ; the particles are slightly coarsely than those in the previous unit. The AMS ^14^C ages at 6.78 m, 11 m, and 15.7 m are 2350 ± 30 a BP, 3090 ± 30 a BP, and 8520 ± 30 a BP, respectively, and the OSL age at 13.55 m was 4.8 ± 0.3 ka. The contents of illite (72.4%) and smectite (8.42%) were lower than those in the upper stage, and the contents of chlorite (8.2%) and kaolinite (11%) are slightly lower than those in the upper stage. The ratios of major elements Al_2_O_3_/SiO_2_, Al_2_O_3_/Na_2_O, and Al_2_O_3_/K_2_O gradually decrease from bottom to top, whereas those of other elements increase slowly. Except for Cu, the contents of trace elements decrease gradually from bottom to top.

U3 (5–0 m): the sediments are mainly gray-yellow medium sand and medium fine sand, with an average particle diameter of 2.1Φ; the particles are coarser than those in the previous stage. The contents of illite (76.4%), kaolinite (12.2%), and chlorite (11.4%) are higher than those in the previous stage; smectite is absent in this stage. The ratios of major elements Al_2_O_3_/Fe_2_O_3_, Al_2_O_3_/MgO, Al_2_O_3_/TiO_2_, and Al_2_O_3_/P_2_O_5_ exhibit an increasing trend, whereas those of other major elements remain essentially unchanged. The contents of trace elements such as Cr, Co, Ni, Cu, Zn, Nb, Ta, Hf, Zr, and Ce are lower than those in the previous stage.

## Discussion

### Applicability of proxies for the East Asian summer monsoon

The sediments of the South China Sea record considerable paleoenvironmental information, which can be used to reconstruct the evolution of the East Asian monsoon history and to study paleo-oceanic evolution^11–13,35,42,43^. Previous studies have found that the ratios of Al/Si, Al/K, Rb/Sr, Al/Ti, and K/Ti can effectively record the intensity of surface chemical weathering ^10,44–47^, which is a reliable proxy for climate change. Numerous studies suggest that the particle size fraction of 2–10 µm is a reliable record of wind dust and river sediment input and is thought to be weakly affected by sea level changes or undercurrents; it can thus be used as a proxy for the East Asian summer monsoon^48–50^. Clay minerals, which are commonly found in marine terrigenous clastic sediments, record the history of climatic changes in cold and warm environments in the source area^51–54^ and play an important role in paleo-environmental reconstruction, paleo-monsoon changes, and sea–land correlation studies^55–58^. Previous studies have found that the clay mineral composition in the late Quaternary sediments of the South China Sea records significant glacial-interglacial cycle changes, and the changes in the smectite/(illite + chlorite) ratio and smectite content can effectively indicate the evolution of the East Asian monsoon. Clay minerals are not affected by global glaciation or interglacial oscillation, and the smectite/(illite + chlorite) ratio can be used as a proxy to reconstruct the intensity of the Southeast Asian summer monsoon^59^.

**Figure 8:**
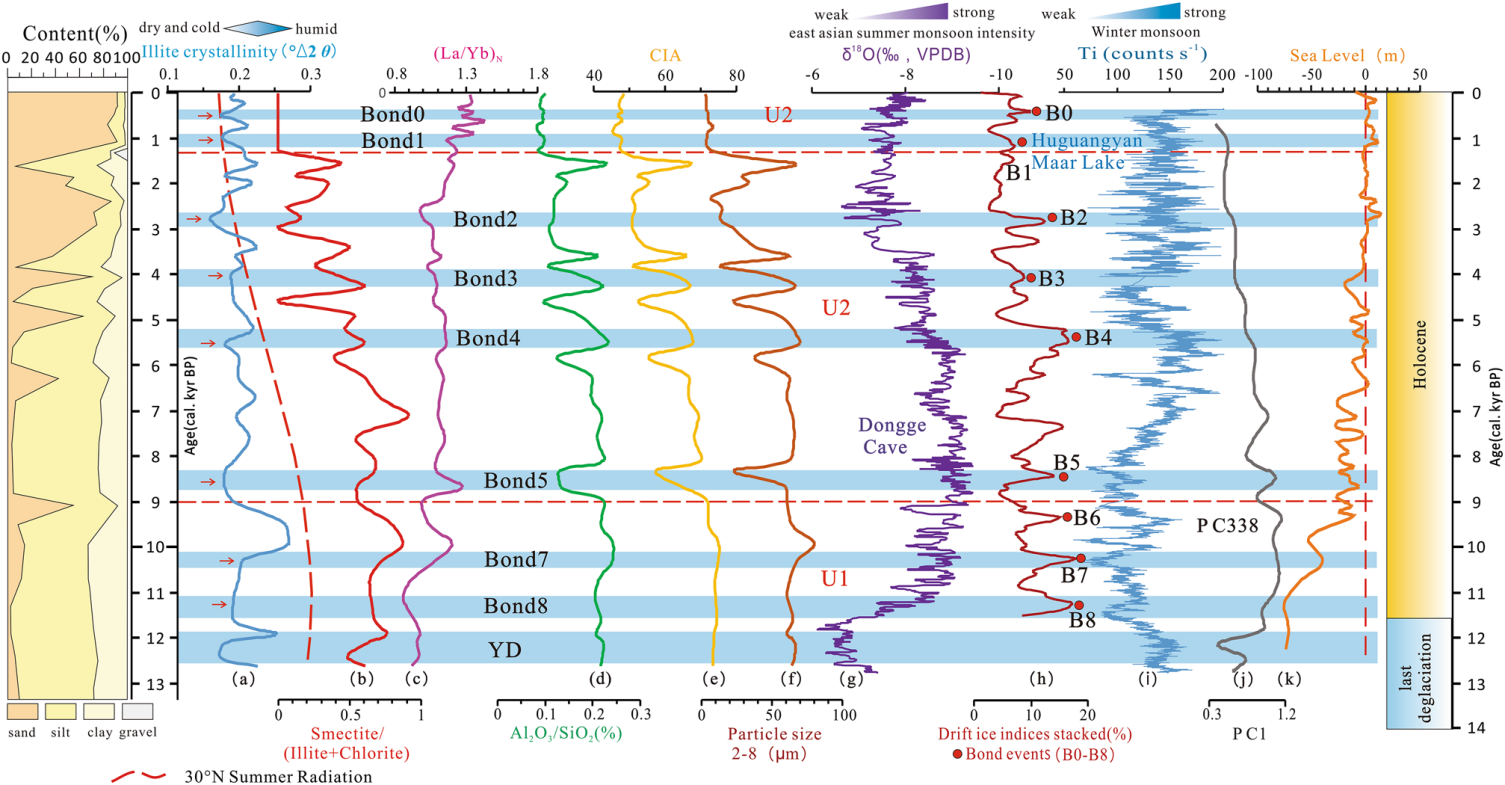
Comparison of elemental ratios with global climate change in core KZK01. Summer sunshine radiation at 30°N, according to Berger and Loutre^65^; (g) Oxygen isotopes of stalagmites in South China, according to Dykoski et al.^24^ and Wang et al.^61^; (h) Drift ice indices stacked, according to Bond et al.^60^; (i) Ti element intensity of Guangdong Huguangyan Maar Lake, according to Yancheva et al.^62^; (j) PC1 of the northern part of the South China Sea slope, according to Li et al.^11^; (k) Relative sea level of the northwestern South China Sea, according to Siddall et al.^66^.

Based on previous research results and the actual conditions in the study area, six weathering parameters, namely illite crystallinity, the smectite/(illite + chlorite) ratio, the (La/Yb)_N_ ratio, the Al_2_O_3_/SiO_2_ ratio, particle size (2–8 μm), and the chemical index of alteration (CIA), were selected for comparison to identify a proxy index suitable for coastal zones reflecting regional surface chemical weathering. The illite crystallinity (Fig. 8a) shows significant reductions at multiple time periods, specifically around 0.52, 1.03, 1.84, 2.78, 4.03, 5.53, 8.33, 10.3, 11.1, and 12.4 kyr BP. These reductions align closely with the Bond events identified by drift ice indices^60^ (Fig. 8h) and the δ^18^O record of stalagmites in Dongge Cave^24,61^ (Fig. 8g), suggesting a correlation between illite crystallinity and climatic variations. This finding indicates that changes in illite crystallinity in coastal sedimentary areas can serve as a potential indicator for understanding and reconstructing past climatic changes, particularly in relation to the East Asian monsoon, as supported by the corresponding Bond events and the Younger Dryas event. Changes in smectite/(illite + chlorite) (Fig. 8b) ratios align with climate records during certain periods (such as YD and Bond events 2 and 5)^24,60,62^, but an inverse correlation has been observed during other cold periods, such as Bond events 0, 1, 3, 4, and 6-8-7. This suggests that this ratio may not be a suitable index to accurately capture the dynamics of these specific cold periods. Studies have shown that rare earth elements have the potential to record climate change, and heavy rare earth elements are more likely to decay in weathering products than light rare earth elements^63^. Thus, a high ratio of (La/Sm)_N_ to (La/Yb)_N_ (chondrite standardization) indicates strong weathering^57^. The (La/Yb)_N_ value (Fig. 8c) decreased slightly during the periods of Bond0, Bond1, Bond2, and Bond8, which corresponded to authoritative climate reconstruction data (Fig. 8g–i). Wei et al. suggested that the (La/Yb)_N_ ratio can reflect the weathering intensity of the source area only when the sediment source is relatively stable^64^. The northwest part of the South China Sea is a large semi-enclosed bay on the continental shelf that is bounded by land on three sides and opens to the South China Sea towards the south (Fig. 1). The sediments in this part of the South China Sea originate from areas such as Taiwan, Vietnam, and the Red River. Owing to the multi-level ocean current system and East Asian monsoon, the hydrodynamic conditions and the source–sink transport deposition process of sediments are very complex^3,13^. The (La/Yb)_N_ index did not significantly reflect cold events such as Bond3 to Bond7, which was possibly due to the low sample resolution and unstable sediment source. The curves for Al_2_O_3_/SiO_2_ (Fig. 8d), CIA (Fig. 8e), and particle size (2–8 μm) (Fig. 8f) are similar, and all three only show a significant decrease during the periods of Bond5, which was inconsistent with climate records. The curves for Al_2_O_3_/SiO_2_ CIA, and particle size (2–8 μm) did not show significantly low values during the cold periods, which may have been caused by the masking of paleoclimate information by provenological and sea level change. Wan et al. selected CIA values for the < 2 μm particle size fraction of records of surface weathering in the Red River basin since about 6400 cal yr BP, as fine-grained sediments can be transported over long distances without being affected by sorting^50^. In this study, only bulk sediment was analyzed for CIA. By comparing our data with the climate reconstruction index PC1^11^ from the continental slope sediments of the South China Sea (Fig. 8j), we found that the climate record index extracted from the coastal sediments is both complete and sensitive to the record of cold events, indicating the great potential of illite crystallinity as an index for the reconstruction of the East Asian monsoon. It also showed the superiority of coastal sedimentary areas as paleoclimate research sites. Thus, illite crystallinity was found to be a good proxy index of East Asian summer monsoon intensity in the northwestern coastal zone of the South China Sea.

### Mechanism driving regional summer monsoon

In previous studies, illite crystallinity was commonly used to indicate chemical weathering. Illite crystallinity was 1 nm based on the half-height peak width, and the high value represented poor illite crystallinity, strong hydrolysis in the terrestrial source area, and warm climate characteristics. Low half-height peak width values represent high illite crystallinity, weak hydrolysis, and dry and cold climate characteristics^34,67,68^. As can be seen from Fig. 8a, illite crystallinity index records Younger Dryas events (YD) and Bond events since the Holocene. This shows that the illite crystallinity index can effectively record regional climate change.

The intensity of the East Asian summer monsoon recorded by the illite crystallinity index was almost synchronized with the widely accepted δ^18^O recorded in the stalagmites in South China. The intensity of the East Asian summer monsoon reflected by illite crystallinity was essentially consistent with the 30° summer solar radiation^65^ in the Northern Hemisphere, indicating that the climate change in the northern part of the South China Sea was driven by the summer solar radiation in the Northern Hemisphere. Evidence of global cold events (YD and Bond et al.) exists in the South China Sea, Tibet, tropical lake Huguangyan maar, and loess regions in China^69–72^, indicating that the climate in East Asia is controlled by the ice sheet at high latitudes. Xu et al. identified YD, Heinrich 1 (H1), Heinrich 2 (H2), Heinrich 3 (H3) and other widespread cold events based on the chemical indexes of the CS11 core from the northern deep basin of the South China Sea^12^. The total organic matter (TOM) data of Huguangyan maar Lake revealed the weakening of the summer monsoon at 9.2 ka, 8.2 ka, 5.3 ka, 4.2 ka, 2.8 ka, 1.4 ka, and 0.4 ka; this trend was consistent with that of global cold events^73^. Li et al. performed principal component analysis on element data from core PC338 in Qiongdongnan Basin in the northwestern part of the South China Sea and found that PC1 (Fig. 8j, representing terrigenous fine-grained sediments) recorded global cold events, including Heinrich events, Younger Dryas, and the 8.2 ka event. The summer light intensity at low latitudes is also thought to drive the summer monsoon in the Red River Basin^11^. Liu et al. found that the kaolinite/(illite + chlorite) ratio off the coast of Vietnam showed obvious glacial-interglacial cycle variations and that the high-frequency variation of smectite content was in good agreement with the summer solar emphases of the Northern Hemisphere at low latitudes, suggesting that the ice sheet at high latitudes and the tropics at low latitudes drove the evolution of the late Quaternary East Asian winter monsoon and summer monsoon, respectively^74^. The findings of this study were highly correlated with a number of surrounding climate reconstruction indicators. The results showed that the evolution of the East Asian summer monsoon was mainly driven by summer solar radiation in the Northern Hemisphere. Cold climate events were globally consistent and may have been related to the global impact of the ice sheet at high latitudes.

### Impacts of the Qiongzhou Strait opening and sea level changes on regional climate

The Qiongzhou Strait acts as a gateway for the exchange of water masses and atmospheric dynamics between the Beibu Gulf and South China Sea. Thus, this ocean passage may have a significant influence on changes in local and regional climate systems. Our physical and chemical records provide valuable insights, revealing an intriguing transition at 9.0 kyr BP from a local and stable stage to a regional and dynamic stage. This transition is evident through changes in grain size (2–8 μm), the CIA, and Al_2_O_3_/SiO_2_ and (La/Yb)_N_ ratios (Fig. 8c–f), which indicate a significant shift in the sediment composition and geochemical characteristics. Importantly, this shift is not limited to our specific study site, but is also observed in core PC338^11^ (Fig. 8j), highlighting its regional significance.

The transition from local to regional climate signatures may have been impacted by the opening of the Qiongzhou Strait, although the exact timing of the opening of the strait is still under debate. Numerous studies have suggested that the opening of the Qiongzhou Strait likely occurred between 11.0 and 8.0 kyr BP^15–18,75,76^, aligning closely with the timing of the observed transition in our records. Furthermore, a significant increase in global sea level by approximately 60 m (Fig. 8k) from 13.0 to 9.0 kyr^66^ may have reinforced water mass exchange and interaction between the Beibu Gulf and the South China Sea through the strait. Changes in physical and chemical records since 9.0 kyr are consistent with regional climatic fluctuations (Fig. 8), which can likely be attributed to the combined effects of the opening of the Qiongzhou Strait and rising sea levels.

## Conclusion

1. Illite crystallinity extracted from coastal sediments effectively records regionally widely recognized Younger Dryas events and Bond events since the Holocene. The results demonstrate that illite crystallinity index can overcome the limitations of lithology and provenological changes that it has great potential as a proxy index of summer monsoon intensity reflecting regional surface chemical weathering. It is feasible to use the coastal zone, which exhibits the most frequent land-sea interaction, as a site for the reconstruction of the East Asian monsoon.
2. Changes in illite crystallinity reflect the degree of surface chemical weathering in the source area, which correspond to the intensity of the East Asian summer monsoon and the 30°N summer radiation variations. The East Asian summer monsoon during the past 13 kyr BP was mainly driven by the insolation at low latitudes in the Northern Hemisphere. Cold climate events are globally consistent and may be related to the global impact of the ice sheet at high latitudes.
3. Our physical and chemical records clearly demonstrate a distinct transition, which coincided with the opening of the Qiongzhou Strait and rising sea levels around 9.0 kyr BP. The combined effects of these phenomena on sedimentary processes highlight the influence of the Qiongzhou Strait and sea level rise on regional climate dynamics (Supplementary Information).

### Supplementary Information

Supplementary Information.

## Data Availability

The datasets generated during and/or analyzed during the current study are available from the corresponding author on reasonable request.
